# Supplementary material for: Are carbon emissions trading and green financial instruments synergistic? -Comprehensive quantitative research based on content analysis
Source: PLoS One. 2024 Mar 7;19(3):e0298601. doi: 10.1371/journal.pone.0298601 (PMC10919601; doi:10.1371/journal.pone.0298601)
Supplement: S1 Data — (ZIP) [file pone.0298601.s001.zip › Data/地级市Z_F工作报告.zip!/╣π╢1⁄2/│▒╓▌╩╨/└┤╫╘╣1⁄2╓┌║┼í╛┬φ┐╦╩2╛▌═°í┐_2013.docx]

各位代表：

现在，我代表市人民政府向大会作工作报告，请予审议，并请市政协委员和其他列席人员提出意见。

2012年工作回顾

2012年，是本届政府的开局之年。在省委省政府和市委的正确领导下，在市人大和市政协的支持监督下，市政府紧紧依靠全市人民，深入贯彻落实科学发展观，认真应对复杂严峻的经济形势，努力克服各种困难和挑战，着力转方式、调结构、扩内需、稳增长、强基础、惠民生，较好地保持了经济社会平稳健康发展，市十四届人大一次会议确定的年度任务基本完成。

全市实现生产总值706.5亿元，比上年增长10.6%，增速居全省第10位。地方公共财政预算收入31.9亿元，增长17.1%。各项税收收入80.6亿元、增长10.8%，其中国税国内税收收入增长8.9%、地税国内税收收入增长12.2%。城镇居民人均可支配收入17645元，增长12.6%。农村居民人均纯收入8889元，增长14.2%。社会消费品零售总额317亿元，增长14%。

过去一年，我们重点做好以下几个方面工作。

一、积极实施投资带动战略

全面落实鼓励投资促进措施，全年实现固定资产投资224.2亿元，增长12.7%。其中，交通、水利、电力、工业分别完成投资16.4亿元、1.9亿元、12.4亿元、141亿元。一方面，通过加强协调服务，落实挂钩督办制度，缓解了用地难、资金难、审批难等突出问题，较好地推进了潮州港进港公路、亚太通用码头一期工程等重点项目建设，年度50个重点项目完成投资83.7亿元，占年度计划96.5%。另方面，根据投融资发展的实际和需要，积极拓宽投融资渠道，利用民资、外资实施砲浮线改建等工程，采用BT形式建设韩江东西溪大桥等项目，与厦门港务集团合作开发建设小红山码头及三百门新港区，引进恒大集团进行投资开发，洽谈引资建设亿都国际商贸城和东方国际茶都等重大项目。与此同时，先后3次举办重点项目动竣工仪式，推进了74个项目落户、建设和投产。加强对外招商引资工作，实际利用外资1.42亿美元、增长12.8%。

二、大力帮扶企业应对挑战

切实加强对企业的服务，积极帮助企业克服困难，实现规模以上工业增加值228.4亿元、增长16.7%。一是支持企业稳定经营。认真贯彻落实中央和省各类扶持政策，出台了促进中小微企业健康发展、加快外经贸转型升级等文件，安排市级扶持企业财政资金3000万元，帮助企业申获上级政策资金3.86亿元。二是助推企业开拓市场。成功举办了广东潮州特色产品（武汉）展销暨经贸洽谈会、广东（潮州）工艺美术精品展，精心组织“广货网上行”活动，实现规模以上工业内销产值684.9亿元、增长23.9%，外贸进出口42.3亿美元、增长1.2%。三是加强对企业的服务。建立重点企业领导挂钩联系服务制度，协调推进外贸、海关、检验检疫、质监、税务、金融等部门提升服务水平，帮助企业解决发展中遇到的突出问题，支持帮助松发、四通等8家企业争取上市融资。四是强化企业用地支撑。加快推进深圳（潮州）产业转移园建设，基本完成径南产业园首期4500亩用地开发，已有复兴机械等23个项目入园建设或通过入园初审；推进市开发区二期B区和临港产业园征地工作，积极为企业集聚发展、转型升级提供新平台。五是大力培育市场主体。新增私营企业1158户、个体工商户10072户，个体工商户升级为企业128户；鼓励发展直接贸易，新增境外投资企业4家，累计达到19家。

三、全力促进产业转型升级

重视调整优化产业结构，着力推动优势特色产业创新发展。一是积极发展特色农业。加强农业生产示范基地和现代农业园区建设，促进生柑、茶叶、花卉、铁皮石斛等特色农业区域化、规模化发展；加快海洋经济特别是“深蓝渔业”发展，深水网箱养殖产业园增加到73公顷。二是提升工业发展水平。积极引导企业加强技术创新，组织企业申报国家、省各类科技项目194项，完成工业技改投资19.7亿元、增长21.1%；新组建省级工程研发中心4个、市级中心7个，新建成省级质检机构2个；深化产学研合作，与中国科学院广州分院等3家院校建立产学研战略合作关系；深入实施知识产权和品牌标准战略，专利申请和授权量明显增加，新增省级名牌产品18项，“庵埠”食品集体商标获得国家批准注册，又有23家企业参与14项国家标准、11项行业标准、5项地方标准的制修订；积极培育发展战略性新兴产业，与省经信委合作共建的新型电子材料产业基地建设正在推进。三是大力发展临港产业。编制实施《潮州港经济区总体规划》，引进亚太码头国际资源物流配送基地等项目，建成欧华能源二甲醚、风电场等产业项目，开展大唐电厂5-6号机组建设前期工作。四是加快发展文化旅游业。编制名城保护规划和古城区控制性详细规划，完成韩祠橡木园建设、己略黄公祠维修工程，启用潮州文物旅游形象标志，申报增加省文物保护单位7项16处，广济桥、龙湖古寨分别入选广东省“南粤十美”和“十大最美古村落”。全年接待游客491.6万人次，实现旅游收入74.8亿元，均比上年增长18%。五是加紧发展生产性服务业。完成粤东水产品物流中心一期工程建设，推进潮州国际陶瓷交易中心、厦深高铁潮汕客运站商贸物流中心等项目的规划建设。

四、努力加强宜居城乡建设

切实加强城乡规划、建设和管理。一是科学规划引导城乡建设。推进中心城区控规全覆盖和村镇规划编制，完成绿道网、户外广告设置等8个专项规划编制；加快实施城市向东发展战略，推进潮州大桥等项目的规划建设，开工建设“恒大城”一期工程，完成东兴北路等截污工程的规划设计。二是积极实施城市畅通工程。修建改造外环北路、枫韩线市区段和枫春路等城市道路，对市区主要道路路灯实施改造提升，提高了城市美化、亮化水平；启动7条未开通的城市规划道路贯通工作，并已完成南较西路贯通建设。三是努力改善城乡发展条件。推进了沟尾溪、内洋南总干、河内湖涝区整治工程，开展引韩济饶供水工程前期工作；新建改建县乡公路44公里、通村公路275公里，实施2012年新建电力工程项目，强化移动、宽带和光纤网络等通信设施建设。四是大力整治城乡环境。加强城市环境卫生管理，拆除违规违章广告设施255处，开展“大清洁•乡村美”专项活动，顺利通过省卫生城市复审。大力开展韩江综合整治，严厉打击违法采砂行为，确保了韩江水环境和堤围安全。五是着力加强环境生态建设。认真落实节能减排措施，污染减排按计划完成，预计单位生产总值能耗下降4.53%；重视生态建设保护，成功创建为省林业生态市，新增一批名镇、名村和生态示范村。六是加快“三旧”改造步伐。加强节约集约用地，积极推进“三旧”改造，批准“三旧”改造项目768个、面积9072亩。

五、着力抓好保障改善民生

加大财政对民生的投入，民生财政支出55亿元，增长25%，占全市公共财政预算支出的70.9%。一是实施积极就业政策。城镇新增就业3.71万人，培训、转移农村劳动力2.1万人和4.25万人，城镇登记失业率2.35%，低于全省平均水平。二是努力扩大社会保险覆盖面。启动城镇居民养老保险试点工作，基本实现城乡居民养老保险制度全覆盖，全市参加养老、失业、工伤、医疗、生育保险人数分别增长7.99%、4.06%、5.89%、8.61%和9.86%。三是强化社会保障工作。提高最低生活保障待遇，全年发放保障金9307万元；扶贫开发成效明显，有劳动能力的贫困户全部实现稳定脱贫；加强住房保障工作，开工建设保障性住房1470套，改造农村低收入家庭危房2805户。四是启动教育创强工作。投入1.81亿元资金，推进义务教育规范化学校、校安工程建设，完成了市职业技术学校搬迁；严格规范招生行为，促进教育公平发展；提升学前教育、高中阶段教育发展水平，高中阶段毛入学率达到88.5%。五是推进文化强市建设。继续完善公共文化体育服务体系，免费开放公共图书馆、文化馆和文化站，新建镇级公共电子阅览室9个、村级100个，完成市区数字电视转换工作，举办了潮州文化香港展演系列活动、广东省民间潮乐大赛总决赛，潮剧首次在国家大剧院献演并取得圆满成功。六是推进医疗卫生发展。深化医疗卫生体制改革，加强基层卫生服务体系建设；抓好市直医院提质扩容，完成潮州医院扩建，推进市中心医院新址规划建设。七是加强人口计生工作。完善人口信息库建设，推进优生惠民工程，启动孕前免费健康检查，人口计生水平提高。八是重视食品药品安全。建立四级食品安全监督网络，推行餐饮食品和药品安全量化分级管理，提高食品药品安全监管水平。十件民生实事基本完成，兑现了向全市人民的承诺。

六、不断推进改革与管理创新

加强改革与创新管理工作，努力完善社会建设管理体制机制。一是积极完善金融服务体系。引进交通银行、汇丰银行在我市设立分支机构，全面完成城信社退市工作，做大土地储备融资平台，全市金融存贷款余额分别增长12.5%和13%。二是大力开展“三打两建”。查处各类“三打”案件7532宗，打掉欺行霸市团伙158个，破获制假售假大案要案226宗，查办“保护伞”239人，有效地改善了发展环境、维护了群众权益。三是扎实推进平安潮州建设。开展“粤安12”等专项行动，严厉打击“两抢一盗”等违法犯罪，维护社会大局稳定；积极化解各类社会矛盾，妥善处置群体性事件；强化安全生产监督管理，提升应急管理工作水平。四是努力加强管理服务工作。启动全国文明城市创建活动。加快转变政府职能，清理规范行政审批事项，启动网上办事大厅建设，积极推进政务公开和网络问政。抓好统计“四大工程”，加强人事、审计、司法、法制、民政、外事侨务、拥军优抚、贸促、物价、粮食、打私、人防、气象、防震、地方志、档案、对台、民兵预备役、老龄、民族宗教、妇女儿童等工作。五是自觉接受人大政协监督。积极配合做好市人大组织的专项工作评议、专题调研和相关视察活动，以及市政协组织的专题视察和专题议政，有效地推动了相关工作的发展。去年，市政府共办理人大代表建议、政协委员提案133件，满意率达100%。

各位代表，回顾过去一年，成绩来之不易。这些成绩的取得，是省委省政府和市委正确领导的结果，是全市人民团结奋斗的结果，是市人大、市政协监督支持的结果，也是驻潮部队、武警官兵、上级驻潮各单位，以及海内外各界人士支持关心的结果。在此，我代表市人民政府表示衷心的感谢并致以崇高的敬意!

我们也清醒看到，我市经济社会发展还存在一些困难和问题。主要是：经济总量较小，发展水平不高；现代产业体系仍不健全，产业转型升级步伐不快；城市化水平相对偏低，资源环境压力较大；保民生促发展支撑能力不足，社会建设管理还需加强；一些部门办事效率不高，工作作风有待改进。这些问题，我们将进一步采取措施加以克服解决。

2013年工作安排

今年是全面贯彻落实党的十八大精神的开局之年，也是实施“十二五”规划承上启下的重要一年。当前，世界经济低速增长态势仍将延续，各种形式的贸易保护主义明显抬头；国内经济下行压力和产能相对过剩的矛盾有所加剧，企业生产经营成本上升和创新能力不足的问题并存，经济发展和资源环境的矛盾仍然突出。但我们面临的有利因素也逐渐增多，美国经济复苏势头有望增强，欧债危机的冲击逐步缓解；国家继续实施积极的财政政策和稳健的货币政策，经济回升向好的基础逐步巩固，省委省政府推动粤东西北地区加快发展政策措施的完善和落实，为我市经济保持平稳较快发展创造了有利环境和条件。

做好今年政府工作，要坚持以科学发展观为指导，全面贯彻落实党的十八大和省委十一届二次全会、市委十三届三次全会精神，紧紧围绕主题主线和加快转型升级、建设幸福潮州的核心任务，以提高经济增长质量和效益为中心，稳中求进、好中求快，攻坚克难、开拓创新，深入推进改革开放，积极扩大投资消费，加快产业结构调整，着力保障和改善民生，全力促进经济社会加快发展，为我省实现“两个率先”作出应有贡献，为建设富裕潮州、美丽潮州、文明潮州、幸福潮州而不懈努力。

综合各方面因素，今年我市经济社会发展的主要预期目标是：生产总值增长10.2%，地方公共财政预算收入增长12.5%，全社会固定资产投资增长14%，外贸进出口增长4%，全社会消费品零售总额增长13%，居民消费价格指数涨幅控制在3.5%以内，人口自然增长率控制在8.07‰以下，城镇登记失业率控制在3%以下，城镇居民人均可支配收入增长11.5%，农村居民人均纯收入增长13.5%。

为实现上述目标，我们将全力以赴抓好四方面工作。

一、加快产业转型升级，全力提升经济发展水平

产业是经济的基础，要围绕构建现代产业体系这个中心，着力加强产业建设，全面推动产业转型升级和创新发展。

（一）积极发展现代农业

改善农业生产条件。以实施省水利“双千工程”为重点，加快中小河流治理和海堤达标加固工程，完成12宗小型水库除险加固任务。全力推进沟尾溪、内洋南总干、河内湖等三大涝区整治，启动归湖涝区整治工程。抓好农田水利重点县和示范镇项目实施工作，加快中小型灌区配套改造、农业综合开发项目建设和中低产田改造。

促进农业产业化发展。围绕“保供增收惠民生、改革创新添活力”的要求，创新农业生产经营体制，激发农业农村发展活力。推进产业化、标准化农业示范基地建设，大力发展现代农业园区，扶持联户经营和专业大户，提高农业产业化水平。加强农产品流通设施建设，推动农产品批发市场升级改造，搞活农产品流通。建立健全农业公共服务体系，加强农业新品种培育，加快农业科技成果转化。

加快调整农业结构。千方百计稳定粮食生产，提高“菜篮子”产品供给保障能力。深入实施农业名牌带动战略，大力发展茶叶、潮州柑、铁皮石斛等特色产品，积极培育无公害农产品、绿色食品和有机食品，着力发展深水网箱养殖产业，鼓励扶持中深海和远洋捕捞，搞好淡水渔业开发。

（二）提升发展优势特色工业

增强产业创新能力。强化企业创新主体地位，支持企业加强技术改造，引进先进技术和设备，组建研发中心、重点实验室，实施重大科技专项，深化产学研合作。完善创新服务支撑体系，建设提升专业镇、特色产业基地和质检中心等综合服务平台，重点推进新型电子材料产业基地、国家食品软包装产品及设备质检中心建设，加强对已建平台的运营管理。

实施品牌标准战略。深入开展质量强市活动，完善政府质量奖励制度，加强区域品牌培育与保护，推进专利申报、知识产权优势企业认定工作，支持企业创建名牌名标、参与各类标准制修订。实施重大人才工程，加快高层次人才引进和技能应用型人才培养，激发各类人才的创造活力和创业热情。

发展壮大产业规模。加快优势特色产业发展，着力抓好骨干企业提速升级，力争规模以上企业、产值超亿元企业有明显增加，努力打造一批产值超10亿元、超50亿元的龙头企业。大力发展战略性新兴产业，培育发展高新技术产业，加快发展电子机电、生物制药、节能环保、新光源等新兴产业集群。

做大做强民营企业。加强对民营企业的协调服务，支持鼓励兼并重组、股份改造，发展一批成长型中小企业，争取有更多企业列入省500家高成长中小企业帮扶范围。出台扶持措施和管理规定，规范对各级各类扶持资金的管理使用，助推企业做大做强。引导企业创新经营理念和管理模式，加快企业上市步伐，全面提升民营企业的竞争力和影响力。

推动园区提质扩容。狠抓园区促建招商工作，积极承接深圳等发达地区的产业转移。全面完成径南产业园一期开发建设，促进落户项目开工建设、竣工投产，启动二期2.23万亩用地规划编制。完善市开发区、高新区管理体制，完成市开发区二期B区征地工作，推进县区产业园区建设，促进园区经济全面发展。

（三）加快发展临港产业

加强基础设施建设。完善进港公路配套设施，推进亚太通用码头深度建设，抓好潮州港公用航道疏浚，以及三百门新港区和西澳港区综合建设。做好马头山片区、堤北片区5000多亩用地征收工作，规划建设港区污水处理厂，加快疏港铁路、引韩济饶供水工程前期工作。启动口岸联检大楼建设，完善口岸各项配套设施。

发展能源石化产业。大力发展临港产业，重点发展能源、石化、装备制造等产业。着力推进大唐发电厂5-6号机组、中石油和中海油投资项目、国际资源物流配送基地、风力发电场等能源石化项目建设，促进明发油脂及配套蛋白生产加工等项目落户。

引导产业集聚发展。完成临港产业园和石化园区控制性详细规划编制，科学有序引导产业发展。强化服务、加强沟通，促进已签约项目尽快投资建设。坚持招商引资与招商选资相结合，争取在引进石油化工、装备制造、水产加工、仓储物流和高端海洋产业上有新成果，加快形成规模化、集聚化发展的临港产业集群。

（四）着力发展文化旅游业

扩大文化旅游影响力。创新旅游宣传推介方式，打响“相约广济古桥、走进潮人故里”宣传主题，突出古城美、体现古城韵，不断提升潮州文化旅游知名度和吸引力。抢抓厦深高铁即将全线开通的有利时机，积极开展旅游推介活动，组织“潮州行”系列宣传，加强区域旅游合作，大力“引客入潮”。

提升旅游发展水平。编制笔架山潮州窑遗址公园规划，完善古城文化旅游区和绿岛山庄、紫莲山庄、东山湖度假村等景区景点的配套建设，支持培育乡村生态文化旅游，发展韩江水上游艇项目。推进恒大五星级酒店和新世纪大酒店建设，加快旅游吃、住、行、游、购、娱等配套建设，完善旅游服务功能，提高旅游经济效益。

推进文化经济融合。鼓励发展文化创意产业，发展壮大木雕、潮绣、手拉壶等工艺美术产业规模。积极培育新型文化产业形态，丰富工艺陶瓷、婚纱晚礼服等产品的文化内涵和旅游元素，用潮文化提升传统特色产业，努力做大文化经济。

（五）大力发展商贸物流业

着力开拓发展国内市场。引导企业把握市场需求导向，及时调整优化产品结构，推进GMC优质制造商审核和GMC新广货标志认证工作，不断提高潮货的知名度和影响力。举办潮州国际陶瓷博览会，积极组织企业参加各类经贸活动，深入推进“广货网上行”，大力发展网上贸易，拓宽企业营销渠道和范围。全面落实国家鼓励消费政策措施，努力扩大消费需求。

积极发展对外经贸合作。落实出口扶持措施，扩大出口信用保险覆盖面，优化通关条件和政务环境，协调做好应对贸易摩擦和反倾销调查工作，引导企业调整出口结构，稳定扩大出口市场。推进陶瓷、服装、食品等外贸出口示范基地建设，培育出口名牌和优势企业，提高品牌带动出口能力。支持鼓励企业“走出去”，深度开拓境外市场，努力发展直接贸易。创新引资方式，推进与国外大型企业合作，提升利用外资的质量和水平。

加快发展高铁港口物流。借助厦深高速铁路潮汕站区与潮汕机场形成的区位优势，做好潮汕高铁经济区的规划及其商贸物流中心的建设，加快潮州陶瓷国际交易中心建设，积极推进建设广东国际陶瓷采购中心，形成陶瓷、食品、不锈钢、旅游商品等专业市场群。依托不断完善的港口基础设施，抓好潮州港三百门港区、西澳港区物流基地规划建设，推动港口物流业发展。

二、加强投资与环境建设，着力增强可持续发展能力

实现科学发展、加快发展和可持续发展，必须坚持投资带动战略，切实加强生态保护和环境建设。

（一）扩大投融资规模效应

推进重点项目建设。强化项目要素保障和组织协调，突破政策处理、项目审批等难点，坚决整治阻挠工程建设不法行为，推进年度57个重点项目建设，确保完成投资90.7亿元。加大协调推进力度，认真解决征地难、资金难等突出问题，加快厦深高铁潮汕火车站、饶平火车站进站道路建设，尽快完善厦深铁路通车运营配套设施；抓好潮惠高速公路潮州段建设，做好漳玉高速公路潮州段建设前期工作；推进护堤公路、铁汫公路和韩江大桥的改造和扩建。加大电网建设改造的组织协调力度，推进220千伏和110千伏输变电工程等电力重点项目建设。

加强招商引资工作。努力引进一批重大产业项目和重要基础设施项目，促进在谈或已签约项目顺利落地建设，鼓励已投产企业增资扩产。深入实施“乡贤回归”反哺工程，争取在外潮籍企业家回乡投资发展。重视做好项目储备和前期研究，努力形成“建成投产一批、开工建设一批、谋划储备一批”的发展格局。积极参与海峡西岸经济区建设，加强与港澳台交流合作，主动融入汕潮揭同城化发展。

促进投融资发展。加强政府投融资平台建设，发挥政府投资的主导作用，鼓励引导各类资本参与城市公共基础设施和社会事业建设。加强政银企合作，优化信贷结构，增加资金投放。加快地方金融发展，稳妥推进农村信用社股份改造，推动小额贷款公司和信用担保机构发展。

（二）提升城市化发展水平

加快韩东新城区建设。围绕城市提质扩容目标，加快完成韩江以东新城区控制性详细规划编制，优化新城区的功能定位和设施布局。做好意东三路周边用地征收工作，加快推进韩江东西溪大桥、潮州大桥建设，做好潮州东大道的规划设计工作，完成穿越恒大城24米规划路建设，加快市中心医院易地新建，协调推进东方国际茶都、亿都国际商贸城，以及潮州恒大城二期等项目的规划建设，全力促进城市向东拓展。

加强城市建设管理。完成枫韩线和外环路市区段改造，统筹做好外环路潮安段建设，畅通城市交通路网。加强城市绿道建设，优化园林绿化体系。完善城市管理机制，加大市政设施建设投入力度，落实城市管理责任，推进市容环境和违章建筑整治。积极推动中石油西气东输项目，实施管道燃气“一张网”建设管理，规范公交、户外广告、房地产等行业管理。

推进新型城镇化发展。统筹考虑产业集聚区和县城发展，形成以城带产、以城促产、产城一体的格局。发挥城市对农村的辐射带动作用，争取潮安撤县设区获得国务院批准，加强城乡交通、水利、电力等基础设施对接，统筹配置教育、医疗、文化等公共资源，促进城乡一体化发展。推进工业进园入区，解决工业无序发展、污染环境等突出问题，走新型工业化城镇化发展道路。加快农村劳动力转移就业，支持符合条件的农村人口落户城镇，为城市化发展提供人力资源支撑。

（三）推进生态文明建设

加强环境生态保护。珍惜背山面海、韩江穿越全境的良好自然生态环境，加强林业生态工程建设和海洋生态修复，加快被破坏山体的复绿，继续抓好韩江、黄冈河等综合治理，严厉打击乱砍滥伐、盗采稀土瓷土和河砂等违法行为，抓好土壤、大气、固体废物和重金属污染防治。大力发展循环经济，推进绿色发展、低碳发展，努力建设美丽潮州。

推进农村环境整治。加快村庄规划编制，推进新农村建设，大力整治农村环境卫生，抓好排涝、排污渠系建设，做好水体、畜禽养殖污染防治和污水处理，建立完善农村垃圾收运体系，积极改善农村人居环境。大力创建名镇名村示范村，建设名村29个、示范村61个。

抓好节能减排工作。出台能源消费总量控制实施方案，推进以陶瓷窑炉为重点的节能降耗技术改造，推广应用节能产品。落实污染减排目标责任制，严格控制主要污染物新增排放量，完成大唐电厂1-3号机组脱硝等改造工作。加快各级污水处理设施及配套管网建设，完成潮枫路排污管道、东兴北路截污管道，以及沙洲岛人工湿地等建设。

促进节约集约用地。创新土地征收储备机制，规范建设用地管理，抓好闲置土地清理处置。争取上级用地指标支持，强化用地规划和年度计划实施管理。加快推进“三旧”改造，提高土地使用效益。加强土地执法检查，坚决打击违法违规用地行为。

三、坚持以民生为重，大力加强社会建设管理

以保障和改善民生为重点，加强和创新社会管理，不断健全基本公共服务体系，推动和谐幸福潮州建设。

（一）着力保障改善民生

加大公共财政投入。加强税收征管工作，规范管理、公平税负，拓宽税基、增加收入。调整优化支出结构，加大对民生领域和社会事业的投入，落实财政强农惠农富农政策，做好新一轮扶贫工作，努力促进基本公共服务均等化。

重视做好就业工作。构建就业服务平台，大力扩增就业岗位，做好高校毕业生、就业困难人员等重点群体的就业工作。加大农村劳动力技能提升培训力度，促进农村劳动力转移就业。加快创业型县（区）建设，鼓励自主创业、自谋职业，推动以创业带动就业。

提高社会保障水平。推进社保扩面征缴。引进商业保险机构参与医保服务，稳步提高医疗保障水平。推进扶贫帮困工作，努力解决特困群众看病、住房、子女上学等问题。落实相关政策，解决好优抚对象、企业军转干部、伤残军人等特殊群体的生活。

抓好十项民生实事。重视纾民生、解民困，集中力量办好民生实事。一是全力促进就业，新增城镇就业2.8万人、下岗失业人员再就业6000人，组织农村劳动力培训1.5万人、转移就业2万人。二是加大社会保障力度，实现城乡居民养老保险制度全覆盖，动工建设市残疾人康复中心大楼。三是实施市绵德小学重建工作，筹建市特殊教育学校，将职业技术教育免学费实施范围扩大至所有农村户籍全日制在校生。四是推进市中心医院易地新建，完成市第三人民医院扩建工程，建成1家以上平价医院。五是抓好文化惠民工作，新建一批镇村公共电子阅览室。六是抓好住房解困保障工作，开工建设城镇保障性住房1000套，改造农村低收入家庭危房2555户。七是加强农村道路建设，新建改建县乡公路50公里、建设通村公路210公里。八是继续推进村村通自来水工程，解决新增11.2万人的农村饮水安全问题。九是完善绿道网示范段配套设施，建设生态景观林带69公里、森林碳汇工程7.68万亩。十是推进7条未开通城市规划道路的贯通建设。

（二）着力发展各项事业

加快教育事业发展。以教育创强为抓手，统筹推进各级各类教育均衡发展，巩固提升高中教育质量，大力发展职业教育，加快规范化乡镇中心幼儿园和村级幼儿园建设，争取枫溪区和13个镇通过教育强区强镇验收。

提高健康保障水平。完善医药卫生体制，推进公立医院改革，巩固基本药物制度，逐步健全城乡医疗卫生服务体系。完善全市卫生信息平台，推进健康卡流通使用。开展爱国卫生运动，抓好疾病控制、卫生监督、妇幼保健、精神卫生等公共卫生工作。健全食品药品安全监管机制，保障人民群众饮食用药安全。

大力建设文化强市。健全公共文化服务设施，推进市图书馆、档案馆和县区数字影院等建设，构建普惠型公共服务体系。促进文化遗产的保护、传承和发展。推进全国文明城市创建工作，广泛开展文化惠民活动，办好市第十五届运动会和饶宗颐国际学术研讨会，丰富群众文化体育生活。

加强人口计生工作。严格控制政策外出生，着力稳定低生育水平；加强免费孕前优生健康检查，完善利益导向机制，推进人口均等化服务；加强全员信息库建设，实现人口计生保先争优目标。

统筹推进各项工作。加强民族、宗教、外事侨务和对台工作，争取与旧金山建立友城关系，重视发展老龄、残疾人、妇女儿童、红十字事业。切实做好国防动员和民兵预备役工作，推进人民防空体系建设。继续做好广播电视、新闻出版、气象、防震、统计、物价、粮食、档案、地方志等工作，促进各项事业全面发展。

（三）着力加强社会管理

深化重点领域改革。完善公共财政体制，盘活国有资产，加强土地储备出让，增加财政收入。推进金融、价格及要素市场改革，健全土地、资本、人力资源、技术等要素市场。培育发展社会组织，推动政府购买公共服务。

深入创建平安潮州。完善综治信访维稳体系，加大社会矛盾纠纷排查调处工作力度。健全立体化社会治安防控体系，推进平安创建和平安工程建设，完善重点项目建设稳定评估机制，加强社会治安专项整治，依法严厉打击刑事犯罪。落实企业安全生产主体责任，加强应急管理、救援体系建设，提高处置突发事件和应对自然灾害能力。

推进“两建”工作。着力构建以政务诚信为先导、覆盖全市的征信系统为主体、诚信文化宣传教育为支撑的社会信用体系，加快构建行政执法、行业自律、舆论监督、公众参与的多元市场监管体系。

四、建设人民满意政府，努力提高服务发展软实力

着眼人民群众的新期待、事业发展的新要求，创新工作思路和举措，努力建设为民、务实、清廉的政府。

（一）坚持科学施政

建立完善决策咨询、论证、听证等制度，加强政府工作制度建设，提高政府工作的科学性、针对性和创造性。严格依照法定权限和程序行使权力，强化行政执法责任落实。建立目标管理责任制，树立求实务实抓落实的导向，整治庸懒散奢等不良风气。

（二）坚持为民执政

恪尽为民之责，多做察民情、知民意、暖民心的工作，多办打基础、利长远、解民忧、惠民生的实事好事。少开会、开短会，少讲话、讲短话，少发文、发短文，集中精力研究解决实际问题。深化行政审批制度改革，加快网上办事大厅建设，提高办事效率和服务水平，努力让人民群众满意。

（三）坚持依法行政

自觉接受人大、政协监督，提高议案、建议和提案办理质量。广泛听取各民主党派、工商联和各人民团体、无党派人士的意见，自觉接受新闻舆论监督和社会公众监督，加强监察、审计等内部监督，确保行政权力依法公正运行。深入推进政务公开，将重大决策、重大投资、行政审批“晒”在阳光下，方便群众监督。

（四）坚持廉洁从政

切实强化廉洁自律意识，认真落实党风廉政建设责任制和领导干部廉洁自律各项规定，坚持用制度管权、用制度管事、用制度管人。加强廉政文化建设和廉政教育，认真落实“一岗双责”，严厉查处各类违法违纪案件，坚决纠正损害人民群众利益的各种不正之风。

各位代表，完成今年全市经济社会发展目标，任务艰巨、责任重大。让我们在省委省政府和市委的正确领导下，解放思想、脚踏实地，凝聚力量、攻坚克难，共同为建设富裕潮州、美丽潮州、文明潮州、幸福潮州而努力！
